# Effects of short-term methionine and cysteine restriction and enrichment with polyunsaturated fatty acids on oral glucose tolerance, plasma amino acids, fatty acids, lactate and pyruvate: results from a pilot study

**DOI:** 10.1186/s13104-021-05463-5

**Published:** 2021-02-02

**Authors:** Thomas Olsen, Bente Øvrebø, Cheryl Turner, Nasser E. Bastani, Helga Refsum, Kathrine J. Vinknes

**Affiliations:** 1 Department of Nutrition, Institute of Medical Biosciences, Domus Medica, University of Oslo, Sognsvannsveien 9, 0372 Oslo, Norway; 2 Department of Sport Science and Physical Education, University of Agder, 4604 Kristiansand, Norway; 3 Department of Pharmacology, University of Oxford, Oxford, OX1 3QT UK

**Keywords:** Methionine restriction, Cysteine restriction, Oral glucose tolerance, SCD, Fatty acids, Pyruvate

## Abstract

**Objective:**

In this 7-day pilot study we randomized healthy, normal-weight men and women to either a dietary intervention with methionine and cysteine restriction enriched in PUFA (Met/Cys_low_ + PUFA, n = 7) or with high contents of methionine, cysteine and SFA (Met/Cys_high_ + SFA, n = 7). The objective was to describe the short-term responses in oral glucose tolerance, amino acid profile, total fatty acid profile, pyruvate and lactate following a Met/Cys_low_ + PUFA diet vs. Met/Cys_high_ + SFA.

**Results:**

The diet groups consisted of five women and two men, aged 20–38 years. After the 7-d intervention median pre- and post-oral glucose tolerance test (OGTT) glucose concentrations were 5 mmol/L and 4 mmol/L respectively in the Met/Cys_low_ + PUFA group. In the Met/Cys_high_ + SFA group, median pre- and post-OGTT glucose concentrations were 4.8 mmol/L and 4.65 mmol/L after the 7-d intervention. The responses in the amino acid profiles were similar in both groups during the intervention with the exception of serine. Fatty acids decreased from baseline to day 7 in both groups. Plasma lactate and pyruvate were similar for both groups with an increase to day 3 before approaching baseline values at day 7.

**Trial registration:**

ClinicalTrials.gov: NCT02647970, registration date: January 6^th^ 2016.

## Introduction

Methionine and cysteine are amino acids mainly found in foods of animal origin [1], and has roles in methylation reactions, protein synthesis and antioxidant production [2]. Dietary restriction of the amino acid methionine and cysteine leads to many beneficial health effects in animals including increased lifespan, favorable changes in body composition and lipogenic gene expression, increased insulin sensitivity, and anti-inflammatory properties [3].

In animal studies, methionine and cysteine restriction reduced activity of the lipogenic enzyme stearoyl CoA-desaturase (SCD1) [4]. Circulating concentrations of cysteine are inversely associated with plasma SCD activity indices in large observational cohorts [5], further suggesting that amino acids may be involved in the regulation of the expression and/or activity of this enzyme in humans. SCD1 activity is also considered a target for supplementation with polyunsaturated fatty acids (PUFA) and intakes of PUFA-rich foods led to reduced plasma activity indices of SCD1 in a human intervention study [6]. Experimental studies with either methionine and cysteine restriction, or PUFA supplementation, share many of same beneficial effects on metabolic outcomes [3, 7–9].

Over the recent years, we have performed and published results from one 7-d and one postprandial pilot study in which we observed effects of a diet restricted in methionine and cysteine and high in PUFAs on plasma sulfur amino acid profile and SCD1 activity indices [10, 11]. Because the pilot studies were the first of its kind we also collected data on a wide range of potential outcomes including oral glucose tolerance, amino acid and total fatty acid profile as well as markers of glucose metabolism including pyruvate and lactate. The aim of the present paper was to systematically describe the response in oral glucose tolerance, amino acid profile, total fatty acid profile, pyruvate and lactate to a short-term dietary intervention with methionine and cysteine restriction plus enrichment of PUFA vs. a diet high in methionine, cysteine and saturated fatty acids (SFA).

## Main text

### Materials and methods

Participants, inclusion and exclusion criteria, study design, collection of demographic variables, anthropometric measures, lifestyle and health-related data have been described in detail elsewhere [10]. In brief, 14 healthy, normal-weight men (n = 4) and women (n = 10) were recruited through the web and social media page of the University of Oslo. The study was a randomized controlled dietary intervention in which participants were randomized to either a dietary intervention with methionine and cysteine restriction enriched in PUFA (Met/Cys_low_ + PUFA, n = 7) or with high contents of methionine, cysteine and SFA (Met/Cys_high_ + SFA, n = 7). The energy content was on average 2000 kcal/d for women and 2500 kcal/d for men. Both diets were vegan-based without meat, fish, eggs, dairy products and certain plant-based foods. The Met/Cys_low_ + PUFA diet contained on average ~ 23.5 g total PUFA (10.9% of total energy [E%]) and 0.93 g methionine and cysteine for women, and ~ 28 g total PUFA (10.6 E%) and 1.19 g methionine and cysteine for men. The Met/Cys_high_ + SFA contained 30.3 g SFA (13.5 E%) and 5.8 g methionine and cysteine for women, and 37.2 g SFA (13.3 E%) and 6.0 g methionine and cysteine for men. A full overview of the diet compositions and a typical daily menu can be found in the Additional file 1. The study protocol was approved by the Regional Committee for Medical Research Ethics South East and was registered with the United States National Library of Medicine Clinical Trials registry (ClinicalTrials.gov Identifier: NCT02647970, January 6^th^ 2016). The study was in accordance with the Declaration of Helsinki.

The oral glucose tolerance test (OGTT) procedure consisted of consumption of 75 g glucose mixed in 4 dl of water after an overnight fast. Blood glucose concentrations were measured 2 h post-OGTT.

Venous blood samples were collected at baseline, days 3 and 7 from each participant after an overnight fast for the amino acids, total fatty acids, lactate and pyruvate. The oral glucose tolerance test was performed at baseline and day 7. Samples were drawn in to EDTA-lined vacuum tubes and immediately centrifuged for five minutes at 4 °C. Plasma aliquots were stored at − 80 °C until analysis. For lactate and pyruvate, a plasma aliquot was immediately precipitated with 5-sulfosalicyclic acid. The supernatant was stored at − 80 °C until analysis.

Total plasma amino acids were measured by LC–MS/MS using a modified version of a previously described method [12]. Briefly, isotopically labelled internal standards were added to plasma, followed by reduction of disulphides using dithioerythritol and then precipitation of the plasma proteins using 5-sulfosalicyclic acid. LC–MS/MS of the supernatant was carried out using a Shimadzu LC-20ADXR Prominence LC system (Kyoto, Japan) coupled to a Sciex QTRAP5500 mass spectrometer with a Turbo V ion source and TurboIonspray probe (Framingham, MA, USA). Chromatographic separation was achieved on a Phenomenex Kinetex Core Shell C18 (100 × 4.6 mm, 2.6 μm) column (Torrance, CA, USA). Plasma concentrations of lactate and pyruvate were determined by LC–MS/MS. Deuterium labelled lactate isotope was used as the internal standard. LC–MS/MS of the supernatant was performed using a Shimadzu LC-20ADXR LC system coupled to a Sciex QTRAP5500 mass spectrometer with Turbo V ion source and TurboIonspray probe (Framingham, MA, USA). Chromatographic separation of the analytes was achieved using a Restek Ultra AQ C18 (100 × 4.6 mm, 3 μm) column.

Plasma total fatty acid concentrations were determined by gas chromatography mass spectrometry (GC–MS) as described previously [10, 13]. Glucose concentrations were determined by colorimetric methods on a Cobas c702 analyzer (Roche Diagnostics International LTd., Rotkreuz, Switzerland) at the Department of Medical Biochemistry (Oslo University Hospital Rikshospitalet, Oslo, Norway).

According to the CONSORT extension statement (Additional file 6) for pilot trials statistical hypothesis testing is actively discouraging because of the inherent uncertainty in data obtained from small samples, which are not powered to detect meaningful treatment effects [14]. In line with this, we present findings in a descriptive manner with means and median as measures of central tendency and the standard deviation and range as measures of dispersion in tables presented in the additional files. In the plots we strive to present individual datapoints in addition to the mean and/or median as a measure of central tendency. Plots were made in R version 4.0.2 (R for statistical computing, Austira, Vienna) using packages “ggplot2” and “ggthemes”.

## Results

In line with the CONSORT extension statement for pilot trials, we did not conduct formal hypothesis tests but report results in a descriptive manner. A flowchart of the participants can be found in Additional file 2. Characteristics of the study participants can be found in Additional file 3. In brief, each group consisted of 5 women and 2 men. Median (range) age was 31 (20, 37) and 24 (21, 38) years in the Met/Cys_low_ + PUFA and Met/Cys_high_ + SFA groups, respectively, whereas body mass index was 22.6 (21.0, 25) and 22.3 (20.7, 26.6) kg/m^2^ at baseline.

**Fig. 1:**
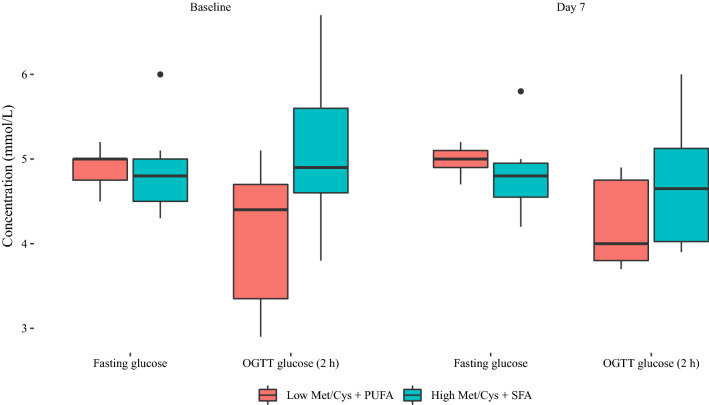
Boxplots showing glucose concentrations before (fasting) and 2 h after an oral glucose tolerance test at baseline and day 7 of the intervention. *Met/Cys,*methionine and cysteine, *OGTT* oral glucose tolerance test, *PUFA* polyunsaturated fatty acids, *SFA* saturated fatty acids

An overview of the metabolite response to the intervention is given in Additional file 4. The response in the OGTT is illustrated in Fig. 1**.** At baseline, median (range) fasting glucose concentrations were 5.0 (4.5, 5.2) and 4.8 (4.3, 6.0) mmol/L, whereas two hours post-OGTT concentrations were 4.4 (2.9, 5.1) and 4.9 (3.8, 6.7) mmol/L in the Met/Cys_low_ + PUFA and Met/Cys_high_ + SFA groups, respectively. After the 7-d intervention, fasting glucose concentrations were 5.0 (4.7, 5.2) and 4.8 (4.2, 5.8) mmol/L, whereas 2 h post-OGTT concentrations were 4.0 (3.7, 4.9) and 4.65 (3.9, 6) mmol/L in the Met/Cys_low_ + PUFA and Met/Cys_high_ + SFA groups, respectively.

**Fig. 2:**
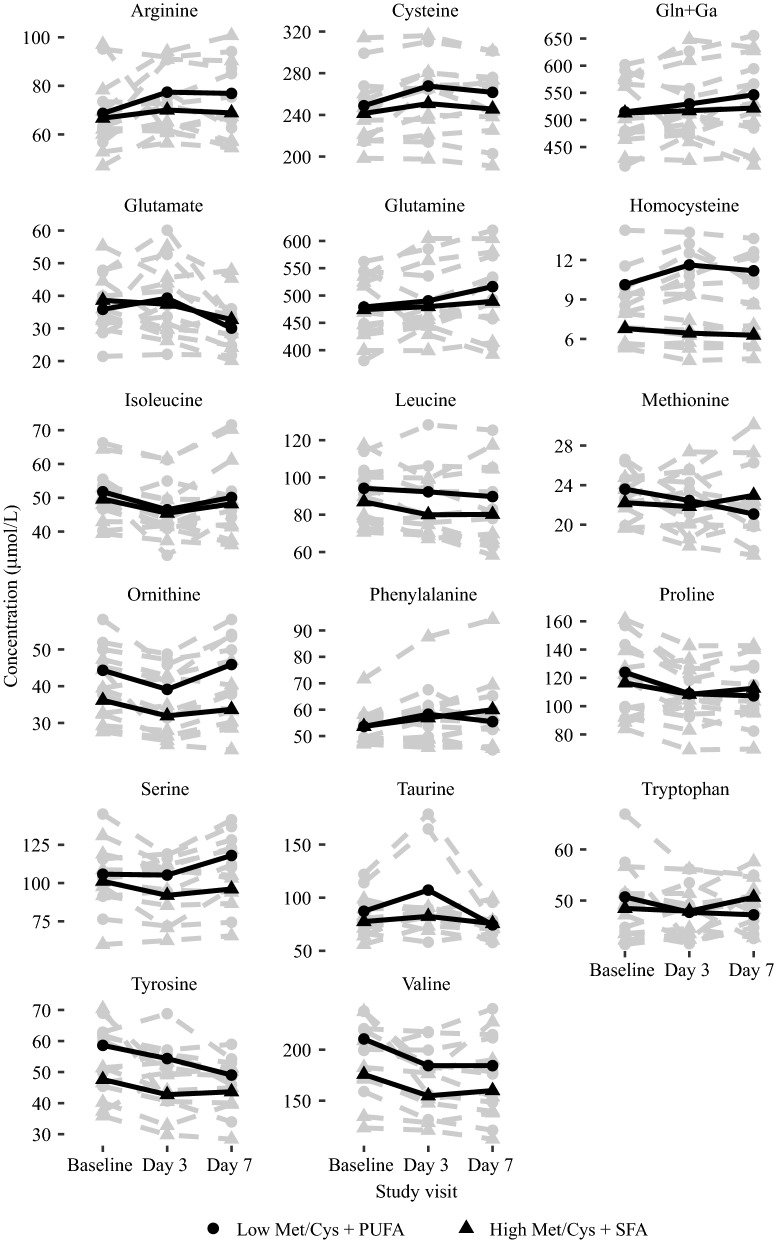
Shows the amino acid concentrations for each individual (grey lines) and the mean concentrations (black lines). *Gln+Ga* glutamine + glutamate, *Met/Cys* methionine and cysteine, *PUFA* polyunsaturated fatty acids, *SFA* saturated fatty acids

The response in plasma amino acid profile are illustrated in Fig. 2. The sulfur amino acid response has been described in a previous publication [10]. For the other amino acids, plasma concentrations responded similarly in both groups throughout the intervention with the exception of serine. Median (range) serine was 103 (76.4, 145) µmol/L in the Met/Cys_low_ + PUFA group at baseline and 123 (74.4, 141) µmol/L at day 7. In the Met/Cys_high_ + SFA group, plasma serine was 101 (59.8, 131) at baseline and 96.3 (65.5, 122) µmol/L at day 7.

**Fig. 3:**
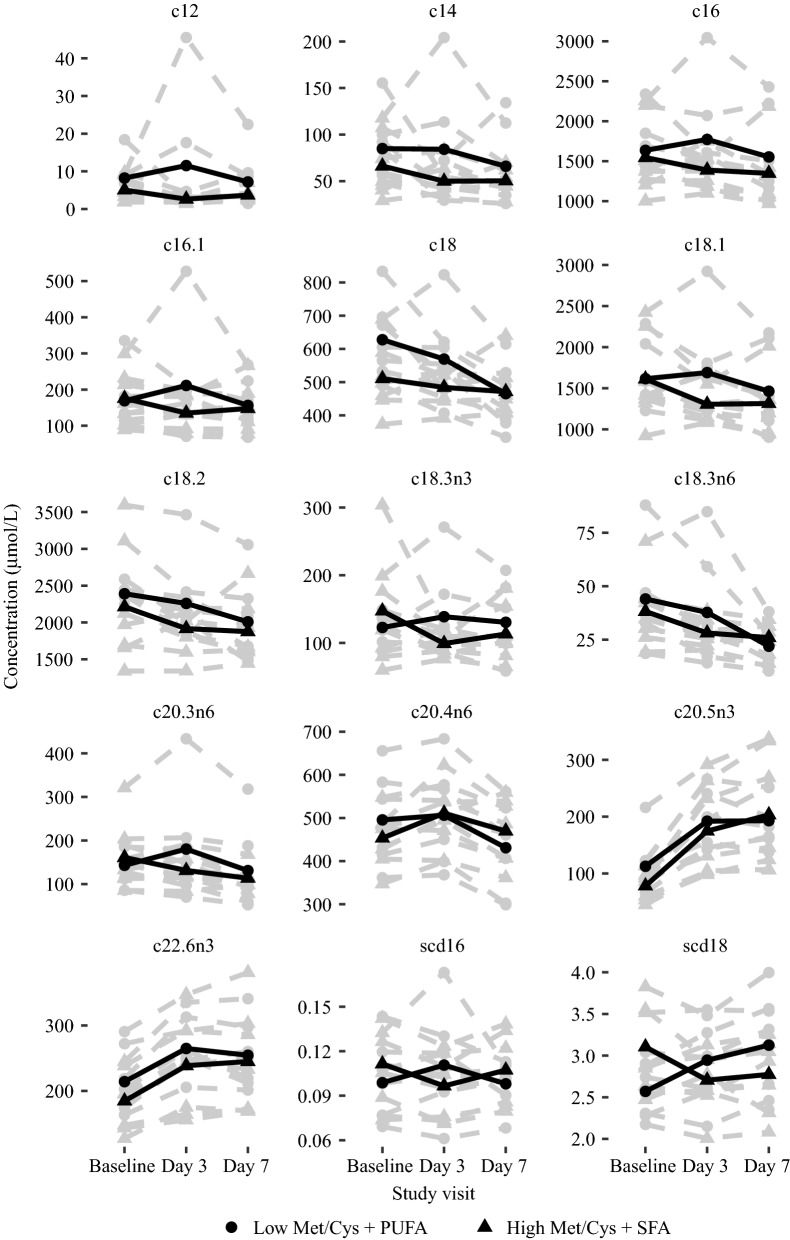
Shows the total fatty acid concentrations for each individual (grey lines) and the mean concentrations (black lines). *Met/Cys* methionine and cysteine, *C12* lauric acid, *C14* myristic acid, *C16:0* palmitic acid, *C16:1* palmitoleic acid, *C18* stearic acid, *C18:1* oleic acid, *C18:2* linoleic acid, *C18:3-n3* α-linoleic acid, *C18:3-n6* γ-linoleic acid, *C20:3-n6* Dihomo-γ-linoleic acid, *C20:4-n6* Arachidonic acid, *C20:5-n3* Eicosapentanoic acid, *C22:6-n3* Cervonic acid

The response in serum total fatty acid profile is illustrated in Fig. 3. The fatty acid response was generally similar in both groups with decreases in most fatty acids from baseline to day 7.

The responses in plasma lactate and pyruvate are illustrated in Additional File 5 and were similar for both groups with an initial increase to day 3 before approaching baseline values at day 7.

## Discussion

Despite the extensive research into sulfur amino acid restriction in animals [3, 9, 15–19] and positive association with adverse health outcomes in human observational studies [5, 20, 21], this has surprisingly not been matched by human interventional studies with a few exceptions [22, 23]. In addition, the potential interaction of sulfur amino acid restriction and PUFA enrichment has not been addressed except for previously published pilot studies from our group [10, 11]. In this pilot study we describe the response in oral glucose tolerance, plasma amino acids and total fatty acid profile, lactate and pyruvate to a short-term diet restricted in the sulfur amino acids methionine and cysteine and enriched in PUFAs. Due to the descriptive nature of the study, we cannot draw firm conclusions but provide data that can aid the planning and design of future studies with respect to metabolic outcomes related to amino acid, fatty acids, insulin sensitivity and glucose metabolism.

Of particular interest is the short-term response in the OGTT where the median glucose concentrations after the dietary intervention were lower in the Met/Cys_low_ + PUFA group (5 mmol/L pre-OGTT, 4 mmol/L post-OGTT) than in the Met/Cys_high_ + SFA group (4.8 mmol/L pre-OGTT, 4.65 mmol/L post-OGTT). We emphasize that this was a pilot study that was not designed to make formal comparisons, however these observations may warrant further investigation when considered in light of experimental data from animals. Notably, mice fed a diet low in methionine and cysteine exhibited lower post-OGTT glucose and insulin compared with control mice fed a high-fat diet [24]. An intraperitoneal glucose tolerance test in mice fed a low cysteine diet showed decreased plasma glucose and insulin responses compared to mice fed a high cysteine diet [25]. Detailed outlines of potential underlying mechanisms can be found in the following references [3, 16, 17, 26, 27]. Data is currently lacking from human observational and experimental studies although one study showed that plasma concentrations of total cysteine were associated with insulin resistance in Hispanic adolescents [28]. To further elucidate the role of the intervention diet on glucose metabolism and insulin resistance, long-term randomized dietary studies in individuals at risk for or with insulin resistance to address potential beneficial effects in prevention and/or treatment.

Due to the combination of methionine and cysteine restriction and PUFA enrichment, we were not able to distinguish potential effects of amino acid restriction from PUFA supplementation. However, effects of PUFA supplementation on glucose metabolism and insulin sensitivity is thought to be minimal as highlighted by a recent review and meta-analysis [29].

We have previously reported findings on sulfur amino acids and selected fatty acids [10]. The other amino acids generally responded similarly with the exception of serine. This is unsurprising because the amino acid composition of the two diets were identical, however, serine is an intermediate of methionine and cysteine metabolism and increased concentrations may be linked to changes in sulfur amino acid metabolism [2].

Changes in fatty acid profile, and pyruvate (which can be produced from cysteine) and lactate were generally similar in both groups, and it is possible that the amino acid and fatty acid interventions were neither intensive nor long enough to induce expected changes [10].

## Limitations

The interpretation of these results is limited by the small sample size, and the short duration of the study and it is unlikely that meaningful effects could be detected during such a short period of time. In addition, there were considerable individual variation in both baseline concentrations and response in the markers, especially for the amino acids and fatty acids, further complicating the interpretation. Future studies can potentially overcome these limitations by enrolling more subjects to increase power. An additional limitation, especially with respect to the clinical relevance of the OGTT data, is that we included healthy normal-weight participants and future studies should address these issues in subjects with, or at risk of developing, metabolic complications. Because this was a descriptive study, we are not able to draw firm conclusions but these data may inform and be useful in the planning and design of future studies.

## Supplementary Information

**Additional file 1:** Contains a typical daily menu and the nutrient contents of the diets.

**Additional file 2:** Contains a figure of the participant flow.

**Additional file 3:** Contains baseline table of the study population.

**Additional file 4:** Contains descriptive statistics of the measured metabolites.

**Additional file 5:** Contains a figure illustrating the data on pyruvate and lactate.

**Additional file 6:** CONSORT checklist.

## Abbreviations

*Met/Cys*_*low*_ + *PUFA*: Diet low in methionine and cysteine and high in polyunsaturated fatty acids
*Met/Cys*_*high*_ + *SFA*: Diet high in methionine, cysteine and saturated fatty acids
*OGTT*: Oral glucose tolerance test
*SCD*: Stearoyl-CoA desaturase

## Availability of data and material

The data included in the current study are available from the corresponding author on reasonable request.
