# Supplementary material for: Effects of short-term methionine and cysteine restriction and enrichment with polyunsaturated fatty acids on oral glucose tolerance, plasma amino acids, fatty acids, lactate and pyruvate: results from a pilot study
Source: BMC Res Notes. 2021 Feb 2;14:43. doi: 10.1186/s13104-021-05463-5 (PMC7852127; doi:10.1186/s13104-021-05463-5)
Supplement: Supplementary file 1 — Additional file 1: Contains a typical daily menu and the nutrient contents of the diets. [file 13104_2021_5463_MOESM1_ESM.docx]

| **Additional File 1 Table 1:** A typical daily menu in the 7-day diet. | |  |
| --- | --- | --- |
| **A typical daily menu** | | |
|  |  |  |
| Breakfast | Oatmeal with apple/raisins or focaccia | Mixed powder with juice |
|  |  |  |
| Lunch | Salad with kidney beans and different fruits and vegetables | Mixed powder with juice |
|  |  |  |
| Dinner | Vegetable casserole/vegetable soup/sweet potato casserole/bean salad | Mixed powder with juice |
|  |  |  |
| Snack | Brazil nuts / fruits | Mixed powder with juice |
|  |  |  |
| 5 capsules of n-3 supplements in the PUFA-Cys/Met diet  2 capsules of n-3 supplements in the SFA+Cys/Met diet | | |

| **Additional File 1 Table 2:** Average nutrient content and composition in the 7-day diet for each intervention group. | | | | |
| --- | --- | --- | --- | --- |
| **Nutrient content** | **Cys/Met_low_ + PUFA** | | **Cys/Met_high_ + SFA** | |
|  | **Women** | **Men** | **Women** | **Men** |
| Kcal | 1987.3 | 2467.6 | 1995.7 | 2504.7 |
| Total fat, *% of energy* | 32.0 | 31.9 | 31.9 | 32.6 |
| Total fat, *g* | 70.6 | 87.3 | 71.4 | 91.3 |
| SFA, *% of energy* | 5.5 | 5.2 | 13.5 | 13.3 |
| SFA, *g* | 12.4 | 14.6 | 30.3 | 37.2 |
| PUFA, *% of energy* | 10.9 | 10.6 | 3.3 | 3.2 |
| PUFA, *g* | 22.9 | 29.0 | 7.4 | 9.0 |
| n-3 PUFA, *g* | 5.2 | 5.9 | 1.5 | 1.8 |
| n-6 PUFA, *g* | 18.3 | 22.1 | 5.0 | 6.2 |
| MUFA, *% of energy* | 12.5 | 13.2 | 11.8 | 12.9 |
| MUFA, *g* | 27.6 | 36.2 | 26.5 | 36.0 |
| Carbohydrates, *% of energy* | 52.7 | 51.7 | 52.0 | 50.7 |
| Carbohydrates, *g* | 262.1 | 319.5 | 262.1 | 319.6 |
| Protein, *% of energy* | 12.2 | 12.7 | 13.1 | 13.3 |
| Protein, *g* | 60.6 | 78.2 | 66.0 | 83.7 |
| Sulfur amino acids, *g* | 1.0 | 1.2 | 5.8 | 6.0 |
| Methionine, *g* | 0.5 | 0.6 | 2.1 | 2.2 |
| Cysteine, *g* | 0.5 | 0.6 | 3.7 | 3.8 |

Data are expressed as means. Abbreviations; MUFA, mono-unsaturated fatty acids; PUFA, polyunsaturated fatty acids; SFA, saturated fatty acids
