# Supplementary figures and images for: Effects of short-term methionine and cysteine restriction and enrichment with polyunsaturated fatty acids on oral glucose tolerance, plasma amino acids, fatty acids, lactate and pyruvate: results from a pilot study
Source: BMC Res Notes. 2021 Feb 2;14:43. doi: 10.1186/s13104-021-05463-5 (PMC7852127; doi:10.1186/s13104-021-05463-5)

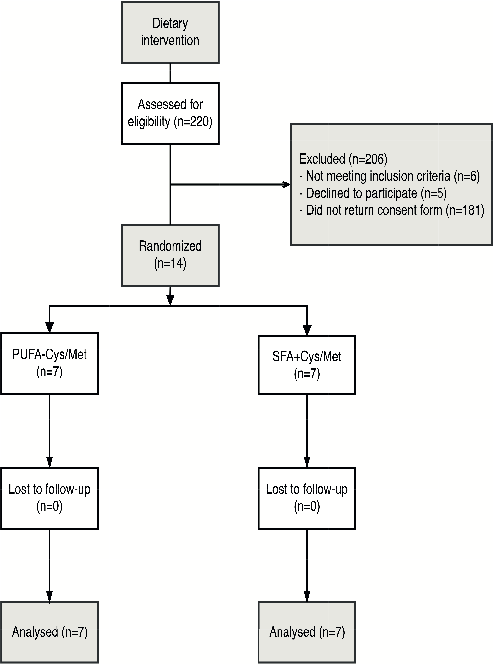
Additional File 2 Figure 1: Participant flow

Supplement: Supplementary file 2 — Additional file 2: Contains a figure of the participant flow. [file 13104_2021_5463_MOESM2_ESM.docx]
