# Supplementary material for: Effects of short-term methionine and cysteine restriction and enrichment with polyunsaturated fatty acids on oral glucose tolerance, plasma amino acids, fatty acids, lactate and pyruvate: results from a pilot study
Source: BMC Res Notes. 2021 Feb 2;14:43. doi: 10.1186/s13104-021-05463-5 (PMC7852127; doi:10.1186/s13104-021-05463-5)
Supplement: Supplementary file 3 — Additional file 3: Contains baseline table of the study population. [file 13104_2021_5463_MOESM3_ESM.docx]

| **Additional file 2:** Characteristics of the study population | | | |
| --- | --- | --- | --- |
|  | **Cys/Met_low_+PUFA** |  | **Cys/Met_high_+SFA** |
|  |  |  |  |
| Female, *n* | 5 |  | 5 |
| Male, *n* | 2 |  | 2 |
| Age, *y* | 31 (20 - 37) |  | 24 (21 - 38) |
| Weight, *kg* | 66.8 (59.2 - 83.9) |  | 65.7 (59.5 - 80.2) |
| Height, *m* | 1.71 (1.66 - 1.89) |  | 1.73 (1.59 - 1.84) |
| BMI, *kg/m^2^* | 22.6 (21.1 - 25.0) |  | 22.3 (20.7 - 26.2) |
| Total cholesterol, *mmol/L* | 4.4 (4.0 - 4.8) |  | 3.8 (2.9 - 5.4) |
| HDL cholesterol, *mmol/L* | 1.6 (1.3 - 2.1) |  | 1.5 (1.2 - 2.3) |
| LDL cholesterol, *mmol/L* | 2.5 (2.0 - 2.6) |  | 2.0 (1.4 - 2.9) |
| Triglycerides, *mmol/L* | 0.7 (0.5 - 1.0) |  | 0.8 (0.4 - 1.0) |

Data are expressed as medians (range) or numbers.
