# Supplementary material for: Effects of short-term methionine and cysteine restriction and enrichment with polyunsaturated fatty acids on oral glucose tolerance, plasma amino acids, fatty acids, lactate and pyruvate: results from a pilot study
Source: BMC Res Notes. 2021 Feb 2;14:43. doi: 10.1186/s13104-021-05463-5 (PMC7852127; doi:10.1186/s13104-021-05463-5)
Supplement: Supplementary file 4 — Additional file 4: Contains descriptive statistics of the measured metabolites. [file 13104_2021_5463_MOESM4_ESM.docx]

**Additional file 3: Mean and median metabolites throughout the intervention^1^**

|  |  | Baseline | Day 3 | Day 7 | Baseline | Day 3 | Day 7 |
| --- | --- | --- | --- | --- | --- | --- | --- |
|  |  | Mean (standard deviation) | | | Median (range) | | |
|  | *Amino acids, umol/L* |  |  |  |  |  |  |
| Met/Cys_low_+PUFA | Arginine | 68.6 (12.9) | 77.4 (10.6) | 76.9 (12.1) | 63.2 (56.8, 95.2) | 75.1 (62.3, 91.8) | 75.4 (62.7, 94.1) |
| Met/Cys_high+_SFA |  | 66.7 (16.9) | 70 (15.6) | 68.9 (18.9) | 62.1 (47, 97.1) | 61.8 (56.5, 94) | 57.4 (54.5, 101) |
| Met/Cys_low_+PUFA | Cysteine | 249 (29.6) | 268 (28.6) | 262 (31) | 249 (215, 299) | 268 (214, 310) | 270 (203, 301) |
| Met/Cys_high+_SFA |  | 241 (38) | 251 (39.7) | 246 (34.9) | 235 (198, 314) | 240 (198, 316) | 244 (191, 301) |
| Met/Cys_low_+PUFA | Glutamate | 35.9 (9.78) | 39.3 (13.4) | 30.1 (5.38) | 34.3 (21.4, 47.9) | 37.1 (22.1, 60.1) | 32.8 (21.8, 36) |
| Met/Cys_high+_SFA |  | 38.6 (8.83) | 37.4 (10.8) | 32.7 (10.4) | 35.9 (29.7, 55.2) | 31.5 (26.2, 55.1) | 29.3 (20.1, 47.8) |
| Met/Cys_low_+PUFA | Glutamine | 479 (70.3) | 490 (52.8) | 516 (71.5) | 449 (381, 562) | 477 (443, 585) | 532 (405, 620) |
| Met/Cys_high+_SFA |  | 474 (52.6) | 480 (75.2) | 489 (78.8) | 469 (399, 535) | 456 (399, 604) | 486 (392, 604) |
| Met/Cys_low_+PUFA | Gln+Ga | 515 (71.4) | 530 (52.6) | 546 (73.9) | 480 (415, 602) | 516 (473, 627) | 566 (430, 656) |
| Met/Cys_high+_SFA |  | 513 (59.6) | 517 (81.6) | 522 (84.9) | 502 (429, 580) | 492 (425, 648) | 515 (416, 633) |
| Met/Cys_low_+PUFA | Homocysteine | 10.1 (2.14) | 11.6 (1.73) | 11.2 (1.7) | 9.39 (8, 14.3) | 11.2 (9.32, 14.1) | 10.5 (8.72, 13.6) |
| Met/Cys_high+_SFA |  | 6.79 (1.51) | 6.44 (1.62) | 6.28 (1.32) | 6.83 (5.31, 9.46) | 6.27 (4.35, 9.38) | 6.46 (4.5, 8.58) |
| Met/Cys_low_+PUFA | Isoleucine | 51.8 (8.11) | 46.5 (9.5) | 50.1 (10.8) | 51.3 (40.2, 66.3) | 43.8 (33, 61.2) | 49.5 (36.7, 71.6) |
| Met/Cys_high+_SFA |  | 49.6 (7.96) | 45.4 (7.93) | 48.2 (12.9) | 49.3 (39.4, 64.3) | 42.9 (37.3, 61.5) | 42 (36.1, 70.3) |
| Met/Cys_low_+PUFA | Leucine | 94.2 (14.4) | 92.3 (20.3) | 89.7 (20.4) | 96.6 (75.4, 114) | 92.4 (69.1, 128) | 83 (62.8, 125) |
| Met/Cys_high+_SFA |  | 86.9 (16.8) | 80 (13.8) | 80.2 (22.3) | 79.7 (70.6, 117) | 74.7 (67.2, 99.7) | 69.8 (58.3, 117) |
| Met/Cys_low_+PUFA | Methionine | 23.6 (2.41) | 22.4 (2.33) | 21.1 (2.75) | 22.7 (19.9, 26.6) | 22.4 (18.5, 25.6) | 20.1 (17.4, 26.3) |
| Met/Cys_high+_SFA |  | 22.2 (2.03) | 21.8 (3.61) | 23 (4.42) | 22.1 (19.6, 24.8) | 20.2 (17.8, 27.4) | 22.3 (16.9, 30.1) |
| Met/Cys_low_+PUFA | Ornithine | 44.3 (10.1) | 39.1 (8.94) | 45.9 (10.7) | 45.3 (28, 58.1) | 41.7 (25.9, 48.7) | 49.9 (29, 58.1) |
| Met/Cys_high+_SFA |  | 36.1 (7.67) | 31.9 (8.04) | 33.7 (7.95) | 32.6 (27.6, 47.3) | 27.9 (24.1, 42.8) | 33.3 (22.7, 46.9) |
| Met/Cys_low_+PUFA | Phenylalanine | 53.6 (3.64) | 58.3 (5.78) | 55.5 (6.3) | 53.9 (48.2, 57.7) | 58.4 (49.8, 67.5) | 55.3 (44.8, 65.2) |
| Met/Cys_high+_SFA |  | 53.7 (8.95) | 56.9 (14.7) | 60 (17.1) | 49.1 (47, 71.6) | 49.3 (45.7, 87.5) | 53.8 (45.3, 94.1) |
| Met/Cys_low_+PUFA | Proline | 124 (29) | 109 (12.9) | 107 (17.1) | 119 (91.2, 159) | 106 (92.5, 127) | 103 (82.3, 129) |
| Met/Cys_high+_SFA |  | 116 (29.1) | 108 (27) | 113 (28.8) | 122 (84, 161) | 104 (69, 143) | 104 (69.6, 143) |
| Met/Cys_low_+PUFA | Serine | 106 (21.3) | 105 (17) | 118 (22.5) | 103 (76.4, 145) | 111 (72, 119) | 123 (74.4, 141) |
| Met/Cys_high+_SFA |  | 101 (22.6) | 92 (19.8) | 96.1 (17.5) | 101 (59.8, 131) | 95.5 (62.4, 112) | 96.3 (65.5, 122) |
| Met/Cys_low_+PUFA | Taurine | 87.2 (23.3) | 107 (45.6) | 74.5 (11.8) | 76.2 (64, 122) | 89.2 (58.1, 179) | 75.1 (57.8, 95.6) |
| Met/Cys_high+_SFA |  | 77.5 (14.8) | 82.4 (8.41) | 75.7 (11.9) | 80.3 (55.9, 98.8) | 84.1 (69.2, 91) | 72.2 (60, 98.9) |
| Met/Cys_low_+PUFA | Tryptophan | 50.7 (8.96) | 47.7 (5.82) | 47.2 (4.42) | 49 (41.4, 66.9) | 48.7 (41.6, 56.2) | 46.1 (42.6, 54.9) |
| Met/Cys_high+_SFA |  | 48.5 (4.84) | 47.9 (4.86) | 50.6 (4.76) | 49 (42.3, 56.6) | 48.1 (42.4, 56.1) | 49.8 (42.7, 57.6) |
| Met/Cys_low_+PUFA | Tyrosine | 58.6 (8.13) | 54.4 (8.6) | 49 (7.94) | 61.4 (45.4, 68.9) | 55.8 (40.7, 68.8) | 48.8 (34, 59) |
| Met/Cys_high+_SFA |  | 47.6 (11.9) | 42.7 (8.86) | 43.7 (8.37) | 46.5 (35.9, 70.4) | 44 (29.8, 53.2) | 44.9 (28.4, 51.4) |
| Met/Cys_low_+PUFA | Valine | 211 (27.5) | 184 (31.6) | 184 (41.3) | 219 (159, 238) | 185 (131, 218) | 178 (121, 240) |
| Met/Cys_high+_SFA |  | 176 (39.7) | 155 (24.4) | 160 (38) | 173 (123, 237) | 152 (121, 181) | 154 (112, 227) |
|  | *Fatty acids, mmol/L* |  |  |  |  |  |  |
| Met/Cys_low_+PUFA | C12 | 8.22 (4.94) | 11.5 (15.9) | 7.19 (7.48) | 7.46 (3.32, 18.4) | 4.48 (2.62, 45.5) | 3.3 (1.48, 22.5) |
| Met/Cys_high+_SFA |  | 5.05 (3.15) | 2.62 (0.766) | 3.69 (1.58) | 4.46 (1.87, 9.63) | 2.71 (1.46, 3.58) | 3.11 (2.37, 6.99) |
| Met/Cys_low_+PUFA | C14 | 84.9 (36.7) | 84.2 (59.3) | 65.9 (41.7) | 83.9 (48, 155) | 71.7 (29.5, 204) | 43.1 (25.9, 134) |
| Met/Cys_high+_SFA |  | 66.2 (34.6) | 49.9 (17.7) | 50.3 (13.6) | 49.4 (29.1, 118) | 45.3 (31.8, 83.4) | 49.3 (27.7, 70) |
| Met/Cys_low_+PUFA | C16 | 1640 (369) | 1770 (624) | 1550 (553) | 1500 (1250, 2340) | 1560 (1160, 3050) | 1310 (999, 2430) |
| Met/Cys_high+_SFA |  | 1550 (491) | 1390 (228) | 1350 (409) | 1370 (1000, 2260) | 1380 (1100, 1750) | 1340 (970, 2180) |
| Met/Cys_low_+PUFA | C16:1 | 169 (86.3) | 211 (147) | 156 (69.6) | 135 (95.8, 335) | 181 (70.9, 527) | 131 (68.2, 269) |
| Met/Cys_high+_SFA |  | 176 (76.1) | 135 (41.1) | 148 (65.2) | 162 (88.9, 299) | 133 (78.3, 187) | 146 (77.2, 267) |
| Met/Cys_low_+PUFA | C18 | 628 (116) | 569 (134) | 465 (102) | 605 (490, 833) | 534 (406, 823) | 489 (334, 615) |
| Met/Cys_high+_SFA |  | 510 (103) | 484 (58) | 471 (83.4) | 497 (373, 689) | 491 (391, 553) | 449 (406, 640) |
| Met/Cys_low_+PUFA | C18:1 | 1620 (394) | 1690 (597) | 1460 (491) | 1420 (1230, 2290) | 1620 (1220, 2920) | 1340 (904, 2170) |
| Met/Cys_high+_SFA |  | 1610 (537) | 1300 (228) | 1310 (359) | 1420 (922, 2420) | 1270 (1080, 1670) | 1360 (940, 2010) |
| Met/Cys_low_+PUFA | C18:2 | 2390 (108) | 2260 (565) | 2010 (555) | 2370 (2240, 2590) | 2040 (1830, 3460) | 1930 (1470, 3060) |
| Met/Cys_high+_SFA |  | 2210 (828) | 1920 (355) | 1880 (426) | 1970 (1340, 3590) | 2020 (1340, 2340) | 1670 (1450, 2670) |
| Met/Cys_low_+PUFA | C18:3-n3 | 123 (22) | 139 (64.8) | 130 (52.1) | 127 (86.9, 149) | 121 (86, 271) | 111 (58.6, 207) |
| Met/Cys_high+_SFA |  | 147 (85.6) | 99.1 (16.4) | 113 (40.7) | 119 (59.8, 304) | 104 (76.3, 120) | 102 (64.8, 181) |
| Met/Cys_low_+PUFA | C18:3-n6 | 44 (21.5) | 37.7 (25.4) | 22 (9.73) | 42.6 (18.6, 88) | 30.5 (14.2, 84.9) | 22.7 (10.4, 38.1) |
| Met/Cys_high+_SFA |  | 38.2 (16.9) | 28.1 (6.49) | 26 (7.57) | 34.7 (19.2, 70.9) | 27.1 (20.3, 38.6) | 28.6 (13.9, 34.5) |
| Met/Cys_low_+PUFA | C20:3-n6 | 143 (37.6) | 180 (121) | 131 (93.5) | 145 (85.8, 189) | 149 (70.3, 433) | 100 (52.7, 318) |
| Met/Cys_high+_SFA |  | 161 (80) | 131 (34.5) | 114 (28) | 133 (85.6, 321) | 120 (83.5, 191) | 116 (78.3, 168) |
| Met/Cys_low_+PUFA | C20:4-n6 | 496 (104) | 506 (111) | 431 (102) | 479 (362, 656) | 501 (368, 684) | 461 (299, 561) |
| Met/Cys_high+_SFA |  | 453 (65.3) | 511 (68.2) | 470 (77.5) | 475 (347, 548) | 510 (401, 623) | 486 (361, 559) |
| Met/Cys_low_+PUFA | C20:5-n3 | 113 (50) | 192 (48.1) | 193 (44.4) | 92.1 (65.8, 216) | 195 (143, 266) | 183 (138, 252) |
| Met/Cys_high+_SFA |  | 78.1 (25.4) | 175 (84.3) | 203 (106) | 85.5 (45, 113) | 131 (98.2, 292) | 146 (106, 337) |
| Met/Cys_low_+PUFA | C22:6-n3 | 214 (49.8) | 265 (44.5) | 255 (50.4) | 203 (163, 291) | 251 (206, 335) | 250 (201, 341) |
| Met/Cys_high+_SFA |  | 185 (46.7) | 239 (75.2) | 245 (82) | 195 (127, 245) | 244 (156, 347) | 233 (169, 381) |
|  | *Glucose metabolism, umol/*L |  |  |  |  |  |  |
| Met/Cys_low_+PUFA | Lactate | 660 (309) | 802 (274) | 686 (111) | 461 (353, 1150) | 811 (473, 1180) | 693 (558, 875) |
| Met/Cys_high+_SFA |  | 710 (218) | 923 (244) | 714 (237) | 654 (396, 1060) | 924 (502, 1220) | 670 (452, 1020) |
| Met/Cys_low_+PUFA | Pyruvate | 55.5 (13.6) | 73.9 (20.3) | 66.5 (9.02) | 56.1 (40.1, 75.6) | 70.4 (53.2, 112) | 65.6 (56.4, 80.6) |
| Met/Cys_high+_SFA |  | 67.7 (24.1) | 88.4 (24.1) | 81.2 (28.9) | 61.3 (43.1, 117) | 91.2 (49.1, 117) | 68 (52.4, 129) |
|  | *OGTT glucose, mmol/L* |  |  |  |  |  |  |
| Met/Cys_low_+PUFA | Fasting glucose | 4.89 (0.248) | - | 4.99 (0.177) | 5 (4.5, 5.2) | - | 5 (4.7, 5.2) |
| Met/Cys_high+_SFA |  | 4.87 (0.568) | - | 4.83 (0.506) | 4.8 (4.3, 6) | - | 4.8 (4.2, 5.8) |
| Met/Cys_low_+PUFA | 2 h glucose | 4.07 (0.879) | - | 4.24 (0.532) | 4.4 (2.9, 5.1) | - | 4 (3.7, 4.9) |
| Met/Cys_high+_SFA |  | 5.11 (0.946) | - | 4.72 (0.818) | 4.9 (3.8, 6.7) | - | 4.65 (3.9, 6) |

^1^Abbreviations: C12, lauric acid; C14, myristic acid; C16:0, palmitic acid, C16:1, palmitoleic acid; C18, stearic acid; C18:1, oleic acid; C18:2, linoleic acid; C18:3-n3, α-linoleic acid; C18:3-n6, γ-linoleic acid; C20:3-n6, Dihomo-γ-linoleic acid; C20:4-n6, Arachidonic acid; C20:5-n3, Eicosapentanoic acid; C22:6-n3, Cervonic acid Met/Cys_low_+PUFA, diet low in methionine and cysteine and enriched in polyunsaturated fatty acids; Met/Cys_high+_SFA, diet high in methionine, cysteine and saturated fatty acids.
