# Supplementary material for: Effects of short-term methionine and cysteine restriction and enrichment with polyunsaturated fatty acids on oral glucose tolerance, plasma amino acids, fatty acids, lactate and pyruvate: results from a pilot study
Source: BMC Res Notes. 2021 Feb 2;14:43. doi: 10.1186/s13104-021-05463-5 (PMC7852127; doi:10.1186/s13104-021-05463-5)
Supplement: Supplementary file 5 — Additional file 5: Contains a figure illustrating the data on pyruvate and lactate. [file 13104_2021_5463_MOESM5_ESM.docx]

Additional file 4 Figure 1

Figure 1: Plasma concentrations of lactate and pyruvate from baseline through day 7 of the dietary intervention.
